# Supplementary material for: What does it take to learn the rules of RNA base pairing? A lot less than you may think
Source: bioRxiv. 2025 Aug 2:2025.07.31.668042. Preprint. [Version 1] doi: 10.1101/2025.07.31.668042 (PMC12324431; doi:10.1101/2025.07.31.668042)
Supplement: Supplement 1 [file media-1.gz › supplemental_material/experiments/g6_optimize_param_conus_rnabench_RNaseP.25.C_uniform/losses_i19.pdf]

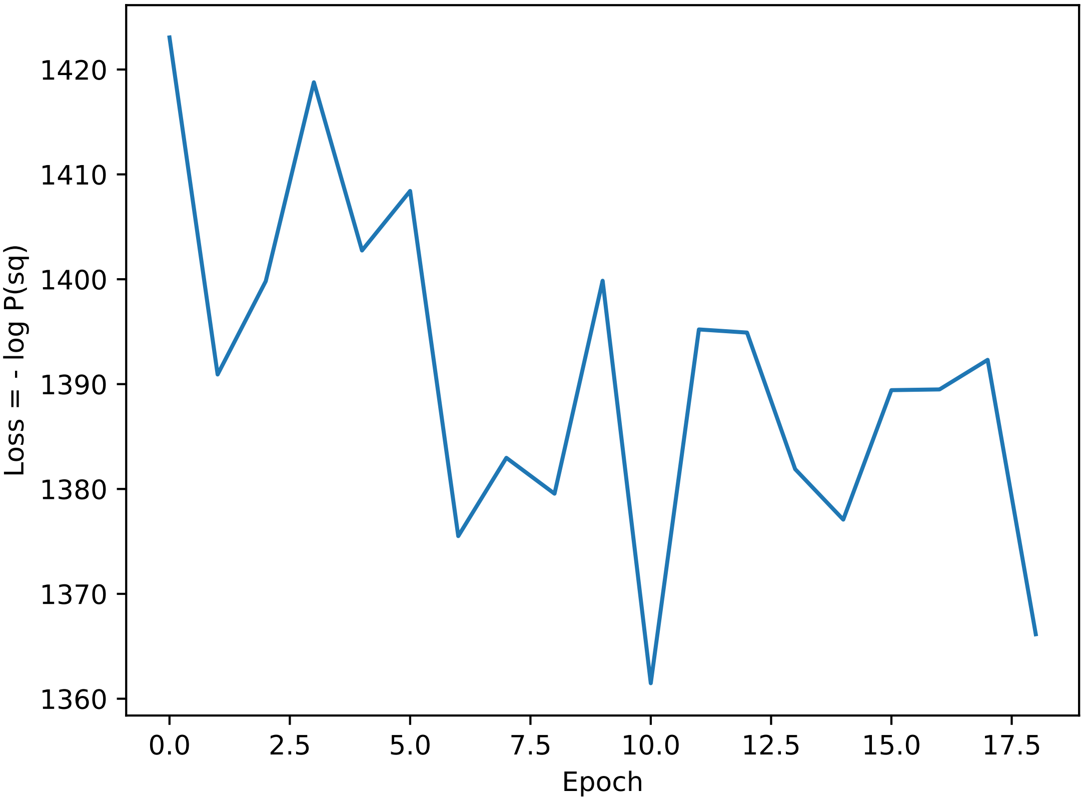

Loss = - log P(sq)
1420
1410
1400
1390
1380
1370
1360
0.0
2.5
5.0
7.5
10.0
12.5
15.0
17.5
Epoch
